# Daily fluctuations in kidney function in critically ill adults

**DOI:** 10.1186/s13054-022-04226-3

**Published:** 2022-11-09

**Authors:** Chao-Yuan Huang, Fabian Güiza, Greet De Vlieger, Geert Meyfroidt

**Affiliations:** 1 Laboratory of Intensive Care Medicine, Academic Department of Cellular and Molecular Medicine, KU Leuven, Leuven, Belgium; 2 Department of Intensive Care Medicine, University Hospitals Leuven, Leuven, Belgium

It is well known that the kidney function can change rapidly during critical illness, with either sudden increases [1] or decreases [2] in renal clearance that may have potentially important consequences for drug dosage adjustments involving renally excreted drugs. However, the true incidence and degree of these fluctuations have never been described systematically. In this study, we aim to investigate kidney function fluctuations as defined by the daily differences in creatinine clearance (CrCl) in critically ill adults.

For the present study, data were retrieved from patients included in the large multicenter EPaNIC randomized controlled trial [3] that compared two parenteral nutrition strategies in 4640 critically ill adults between 2007 and 2010. The Ethics Committee of University Hospitals Leuven approved the use of these patient data for additional analyses (S50404). Daily CrCl was calculated by multiplying the urinary creatinine (UCr) (measured on 24-h urine collection) with the 24-h urine output (UO), divided by the serum creatinine (SCr) times the collection time (1440 min), without correcting for body surface area (BSA).

$$\mathrm{CrCl }(\mathrm{ml}/\mathrm{min})= \frac{\mathrm{UCr}\times \mathrm{UO}}{\mathrm{SCr}\times 1440}$$

As the University Hospitals Leuven were the only EPaNIC center where CrCl was calculated daily, patients from other centers were excluded. Laboratory results were exported from patient data management system database (Microsoft SQL Server®; Microsoft®, Redmond, Washington, USA), and the remaining data were retrieved from EPaNIC trial database (Filemaker Pro®; FileMaker Inc, FileMaker International). As a measure of daily fluctuations in CrCl, we calculated the difference between the CrCl of each pair of two consecutive days. In case the absolute difference in CrCl of 2 consecutive days was less than 20 ml/min, the daily fluctuation was labeled as ‘stable.’ A difference larger than 20 ml/min was defined as ‘unstable.’ We decided to use 20 ml/min as a cutoff as this is a meaningful difference for drug dosing and because the CrCl variability in healthy volunteers has been reported with mean differences of 21.7 ml/min/1.73m^2^ and variations of 18.7% [4]. Depending on the direction of the fluctuation, we used the categories ‘unstable-upward’ if the CrCl on the next consecutive day was > 20 ml/min higher than the previous day, or ‘unstable-downward’ if the CrCl on the next consecutive day was > 20 ml/min lower compared to the day before. We also investigated the relative change in CrCl, defining a relative instable CrCl as a change of > 20% between two consecutive CrCl values. Finally, we examined CrCl fluctuations in the first week of ICU admission separately.

Of the 4389 patients in the study cohort, 2825 patients, corresponding to 18,494 patient-days, met the inclusion criteria (Additional file 1: Fig S1). Descriptive statistics are available in Additional file 1: Table S1. In terms of absolute instability (Fig. 1a), CrCl remained stable in 65% of days, while 19% were unstable-upward and 16% were unstable-downward. Across the CrCl range, the percentage of stable days decreased approximately linearly with increasing CrCl values, such that stability was above 70% for CrCl below 75 ml/min and around 30% for CrCl above 180 ml/min. Additionally, more than 50% of the days were unstable-downward for CrCl above 180 ml/min, and the percentage of unstable-upward days was 20% for CrCl in a range of 45–180 ml/min. When we used the relative definition of a 20% difference (Fig. 1b), the percentages of stable, unstable-upward, and unstable-downward days were 58%, 25%, and 17%, respectively, with an overall CrCl stability ranging around 60% throughout the CrCl range. The percentage of unstable-upward cases declined roughly linearly across the CrCl range, ranging from more than 30% for CrCl below 60 ml/min to about 6% for CrCl beyond 180 ml/min. Additionally, for CrCl below 180 ml/min, the percentage of unstable-downward cases remained low, around 15%, and it rose to 37% for CrCl above 180 ml/min. For absolute and relative differences alike, more instability was observed in the first week of ICU stay (Fig. 1c and d). Specifically, for patient-days within the first week of ICU admission, 39% were unstable, including 23% unstable-upward and 16% unstable-downward days for absolute difference, and 50% were unstable, including 31% unstable-upward and 19% unstable-downward days for relative difference.

**Fig. 1:**
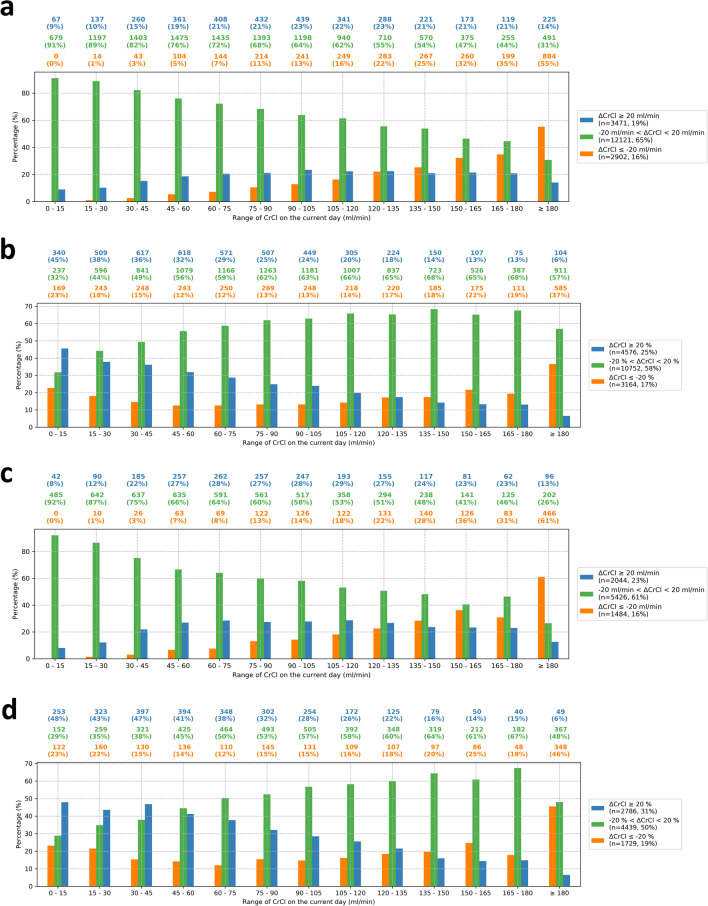
Percentage of patient-days during the entire ICU stay (**a**, **b**) and within the first week of ICU admission (**c**, **d**) with stable, unstable-upward, or unstable-downward CrCl for different CrCl ranges. The number and percentage of patient-days for each CrCl ranges are indicated above the figure. **a, c** the blue, orange, and green bars represent, respectively: an increase larger than 20 ml/min in the CrCl of the next day compared to the current day (*unstable-upward*), a decrease larger than 20 ml/min in the CrCl of the next day compared to the current day (*unstable-downward*), and an absolute difference smaller than 20 ml/min between the CrCl of the next and the current day (*stable*). ΔCrCl, CrCl of the next day minus CrCl of the current day; **b, d** the blue, orange, and green bars represent, respectively: an increase larger than 20% in the CrCl of the next day compared to the current day (*unstable-upward*), a decrease larger than 20% in the CrCl of the next day compared to the current day (*unstable-downward*), and an absolute difference smaller than 20% between the CrCl of the next and the current day (*stable*). ΔCrCl, (CrCl of the next day-CrCl of the current day)/(CrCl of the current day); CrCl, creatinine clearance

To conclude, our findings confirm that potentially clinically significant changes in kidney function may occur on a daily basis in critically ill patients on approximately 35–40% of days, depending on the definition of instability. This instability mainly occurs in the first week of ICU admission and is more pronounced when the CrCl is higher. The measured CrCl is known to overestimate the glomerular filtration rate (GFR) as compared to inulin clearance, but it has been shown the most reliable and cheap method to assess the GFR on a daily basis in the ICU [5]. While these findings have to be confirmed in independent cohorts of critically ill patients, additional investigations are needed to determine the factors associated with fluctuations in renal clearance.

## Supplementary Information

**Additional file 1**. Electronic Supplementary Material: patient cohort characteristics.

## Declarations

### Availability of data and materials

The datasets generated and/or analyzed during the current study are not publicly available due to no prior agreement with the ethical committee but are available from the corresponding author on reasonable request.
