# Supplementary material for: Daily fluctuations in kidney function in critically ill adults
Source: Crit Care. 2022 Nov 9;26:347. doi: 10.1186/s13054-022-04226-3 (PMC9644484; doi:10.1186/s13054-022-04226-3)
Supplement: Supplementary file 1 — Additional file 1. Electronic Supplementary Material: patient cohort characteristics. [file 13054_2022_4226_MOESM1_ESM.pdf]

Daily fluctuations in kidney function in critically ill adults

Chao-Yuan Huang[1], Fabian Güiza[2], Greet De Vlieger[1, 2], Geert Meyfroidt[1, 2*]

[1] Laboratory of Intensive Care Medicine, Academic Department of Cellular and Molecular Medicine, KU Leuven, Leuven, Belgium

[2] Department of Intensive Care Medicine, University Hospitals Leuven, Leuven, Belgium

Running headline: Creatinine clearance may fluctuate significantly on a day-to-day basis.

---

* Corresponding author:

Email: geert.meyfroidt@uzleuven.be (GM)

Supplementary Figures

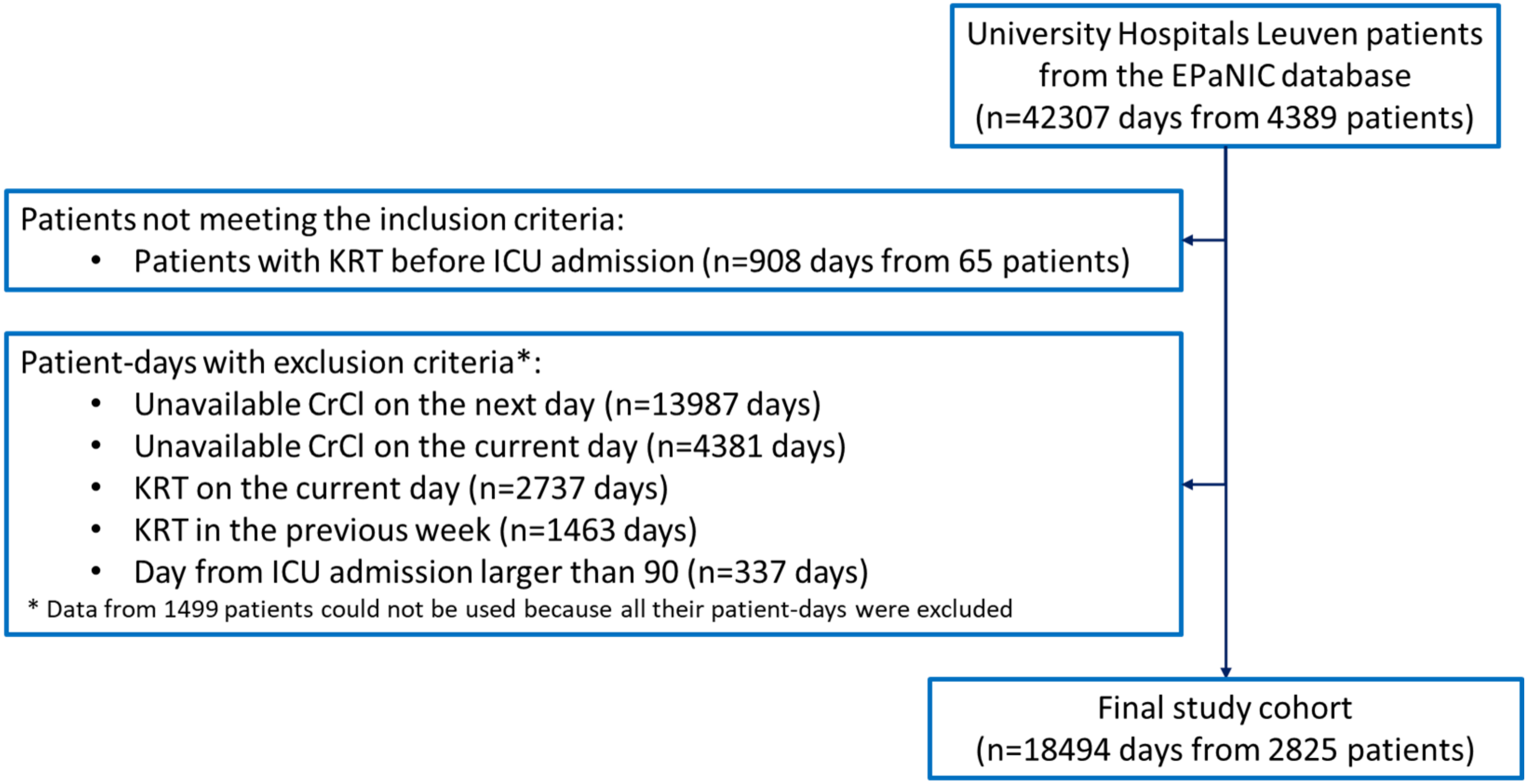

Supplementary Fig. 1 Study cohort. CrCl, creatinine clearance; KRT, kidney replacement therapy; ICU, intensive care unit.

Supplementary Tables

Supplementary Table 1 Patient characteristics and clinical outcomes

	Study cohort (n=2825)
Age, years, median (IQR)	67.59 (56.16 – 75.61)
Gender male, number (%)	1747 (61.84)
Emergency admission, number (%)	1272 (45.03)
APACHE II score, median (IQR)	22 (16 – 32)
Reason for admission	
Cardiac surgery, number (%)	1655 (58.58)
Medical disease, number (%)	114 (4.04)
Neurology and neurosurgery, number (%)	119 (4.21)
Trauma and other surgery, number (%)	662 (23.43)
Transplantation, number (%)	275 (9.73)
ICU mortality, number (%)	162 (5.73)
Length of stay in ICU, days, median (IQR)	5 (3 – 11)

APACHE II score, Acute Physiology and Chronic Health Evaluation II score; IQR, interquartile range; ICU, intensive care unit
